# Reimagining Dental Education: Integrating Digital Technologies for a Global, Digitally Native Generation—A Perspective

**DOI:** 10.1055/s-0046-1823144

**Published:** 2026-06-20

**Authors:** Ulla Palotie, Mikko Liukkonen, Peter Lingström, Szabolcs Felszeghy, Outi Huhtela, Mohammed Al-Haroni, Andreea Didilescu, Simona Georgiana Schick, Damiano Pasqualini, Khaled Ahmed, Anthony J. Smith, Łukasz Zadrozny, Gulsun Gul, Barbara Kispélyi, Bekhzod Yarmukhamedov, Rachid Ait Addi, Benjamin Huberson, Daniela Alejandra Pino Valenzuela, Deepika Kapoor, Edgar Quenta-Silva, Gideon K. Helegbe, Hala Ragab, Hany Mohamed Aly Ahmed, Hilal Aktaş, Kaan Orhan, Katalin Nagy, Kinga Bágyi, Laurah Turner, Latifa Berrezouga, Laura Gartshore, Leonardo M. Nassani, Magrur Kazak, Mahmoud Bakr, Małgorzata Ponto Wolska, Maria Florencia Sittoni Pino, Masako Nagasawa, Mengwei Pang, Mihaela Pantea, Nikunj Sondagar, Nino Tebidze, Thanaphum Osathanon, Peter Hermann, Priyanka Gudsoorkar, Punnya Angadi Rao, Rita Marincsák, Samantha Bryne, Sibel Dincer, Ulf Örtengren, Zohaib Khurshid, Sompop Bencharit, Barry Quinn, Gianrico Spagnuolo, Margaret J. Cox, Mutlu Özcan, Hiroe Ohyama, David P. Rice, Reinhard Chun Wang Chau

**Affiliations:** 1 Department of Oral and Maxillofacial Diseases, University of Helsinki and Helsinki University Hospital, Helsinki, Finland; 2 Department of Ophthalmology, Institute of Clinical Medicine, University of Eastern Finland, Kuopio, Finland; 3 Department of Cariology, Institute of Odontology, Sahlgrenska Academy, University of Gothenburg, Gothenburg, Sweden; 4 Institute of Dentistry, University of Eastern Finland, Kuopio, Finland; 5 Oral and Maxillofacial Teaching Unit, Kuopio University Hospital, Kuopio, Finland; 6 Department of Oral Biology, Faculty of Dentistry, University of Oslo, Oslo, Norway; 7 Department of Clinical Dentistry, Faculty of Health Sciences, UiT The Arctic University of Norway, Tromsø, Norway; 8 Department of Embryology and Microbiology, Faculty of Dentistry, Carol Davila University of Medicine and Pharmacy, Bucharest, Romania; 9 Department of Conservative Dentistry, Clinic for Oral, Dental and Maxillofacial Diseases, Heidelberg University Hospital, Heidelberg University, Heidelberg, Germany; 10 Department of Surgical Sciences, Centro Interdipartimentale di Ricerca Dental School, University of Turin, Turin, Italy; 11 Melbourne Dental School, The University of Melbourne, Victoria, Australia; 12 Department of Oral Biology, University of Birmingham, Birmingham, United Kingdom; 13 Department of Dental Propaedeutics and Prophylaxis, Faculty of Dental Medicine, Medical University of Warsaw, Warsaw, Poland; 14 American Dental Education Association (ADEA), Chicago, Illinois, United States; 15 Faculty of Dentistry, Semmelweis University, Budapest, Hungary; 16 Department of Global Relations and University Administration, EMU University, Tashkent, Uzbekistan; 17 Department of Biology, Cadi Ayyad University, Marrakech; 18 Senior Dentist Clinic, Marrakech, Morocco; 19 Department of Dentistry, Faculty of Health, University of Rouen Normandy, Rouen, France; 20 Facultad de Odontología, Universidad de Concepción, Concepción, Chile; 21 Department of Pediatric and Preventive Dentistry, Luxmi Bai Institute of Dental Sciences and Hospital, Punjab, India; 22 Facultad de Estomatología, Universidad Peruana Cayetano Heredia, Lima, Peru; 23 Department of Biochemistry and Molecular Medicine, University for Development Studies (UDS), Tamale, Ghana; 24 Division of Restorative Dentistry, Department of Clinical Sciences, College of Dentistry, Princess Nourah bint Abdulrahman University, Riyadh, Saudi Arabia; 25 Faculty of Dentistry, Universiti Malaya, Kuala Lumpur, Malaysia; 26 Ankara University Faculty of Dentistry, Ankara, Türkiye; 27 Faculty of Dentistry, University of Szeged, Szeged, Hungary; 28 Faculty of Dentistry, University of Debrecen, Debrecen, Hungary; 29 College of Medicine, University of Cincinnati, Cincinnati, Ohio, United States; 30 Faculty of Dental Medicine, University of Monastir, Monastir, Tunisia; 31 School of Dentistry, University of Liverpool, Liverpool, United Kingdom; 32 Division of Restorative and Prosthetic Dentistry, The Ohio State University College of Dentistry, Columbus, Ohio, United States; 33 Department of Restorative Dentistry, Bahçeşehir University School of Dental Medicine, Istanbul, Türkiye; 34 School of Medicine and Dentistry, Griffith University, Queensland, Australia; 35 Faculty of Health Sciences, Department of Dentistry, Universidad Europea de Valencia, Valencia, Spain; 36 Division of Bio-Prosthodontics, Faculty of Dentistry and Graduate School of Medical and Dental Sciences, Niigata University, Niigata, Japan; 37 College of Stomatology, Chongqing Medical University, Chongqing, China; 38 Faculty of Dentistry, Carol Davila University of Medicine and Pharmacy, Bucharest, Romania; 39 Note Dr., London, United Kingdom; 40 School of Dental Medicine, BAU International University, Batumi, Georgia; 41 Center of Artificial Intelligence and Innovation, Center of Excellence in Dental Stem Cell Biology, and Dental Education Unit, Faculty of Dentistry, Chulalongkorn University, Bangkok, Thailand; 42 Faculty of Dentistry, Semmelweis University, Budapest, Hungary; 43 Department of Environmental and Public Health Sciences, College of Medicine, University of Cincinnati, Cincinnati, Ohio, United States; 44 Department of Oral and Maxillofacial Pathology, Karnataka Lingayat Education Vishwanath Katti Institute of Dental Sciences, KLE Academy of Higher Education and Research, Belagavi, Karnataka, India; 45 Faculty of Dentistry, University of Debrecen, Debrecen, Hungary; 46 Melbourne Dental School, The University of Melbourne, Victoria, Australia; 47 Department of Biomedical Sciences, USC Herman Ostrow School of Dentistry, Los Angeles, California, United States; 48 Department of Cariology, Institute of Odontology, Sahlgrenska Academy, University of Gothenburg, Gothenburg, Sweden; 49 Department of Materials Science and Technology, Faculty of Odontology, Malmö University, Malmö, Sweden; 50 Department of Advanced Free Radical Science, Tohoku University Graduate School of Dentistry, Sendai, Japan; 51 Department of Prosthodontics and Dental Implantology, College of Dentistry, King Faisal University, Al Ahsa, Saudi Arabia; 52 Department of Anatomy, Faculty of Dentistry, Center of Artificial Intelligence and Innovation (CAII), Center of Excellence for Dental Stem Cell Biology, Chulalongkorn University, Bangkok, Thailand; 53 Department of Oral and Maxillofacial Surgery, Biruni University, Istanbul, Türkiye; 54 Department of Restorative Sciences, Adams School of Dentistry, University of North Carolina at Chapel Hill, Chapel Hill, North Carolina, United States; 55 Department of Neuroscience, Reproductive and Odontostomatological Sciences, University of Naples, Naples, Italy; 56 Faculty of Dentistry, Oral and Craniofacial Sciences, King's College London, London, United Kingdom; 57 Clinic of Masticatory Disorders and Dental Biomaterials, Center for Dental Medicine, University of Zurich, Zurich, Switzerland; 58 Department of Restorative Dentistry and Biomaterials Sciences, Harvard School of Dental Medicine, Boston, Massachusetts, United States; 59 Helsinki University Hospital, University of Helsinki, Helsinki, Finland; 60 Faculty of Dentistry, University of Hong Kong, Hong Kong Special Administrative Region (SAR), China

**Keywords:** artificial intelligence, education, dental computer, simulation, virtual reality, health equity, interprofessional education

## Abstract

Digital technologies, such as artificial intelligence (AI), virtual reality (VR), and haptic simulation, are rapidly reshaping oral health education. Traditional phantom head and clinic-based teaching remain essential but no longer suffice alone for digital-native learners or for regulators who increasingly view digital competence as mandatory. Post-COVID evidence suggests immersive and hybrid approaches can enhance motivation, deepen learning, support well-being, and strengthen clinical competence when thoughtfully embedded into curricula. Done well, this integration can foster safer, patient-centered digital care.

Sustainability concerns around traditional, resource-intensive methods are also growing; digital tools can reduce material waste and resource use, aligning dental education with the United Nations 2030 Agenda for Sustainable Development. However, access to these innovations is uneven, raising concerns about a digital divide in graduate readiness and patient care. Networks such as the American Dental Education Association (ADEA), the Association for Dental Education in Europe (ADEE), the Haptic and AI Digital Education Network for Excellence and Research (HAiDENERS) show how collaboration and benchmarking can democratize advanced simulation. Together with active communication involving global research bodies and industry, these developments enable a hybrid, evidence-informed, and equitable training ecosystem whose ultimate goal is improved, safer, and more predictable oral health care worldwide.

## A Pivotal Moment for Dental Education

Due to the rapid progression of digital technology, dentistry requires ambitious thinking and global action. As in other health professions, the integration of artificial intelligence (AI), virtual reality (VR), and haptic technologies has moved beyond experimentation. 1 2 These technologies are beginning to reshape the preparation of future oral health professionals for safe, precise, collaborative, and patient-centered care worldwide. 3 4 5 Dental education stands at a pivotal crossroads where trusted traditional methods meet an expanding digital frontier. In navigating this transformation, careful consideration must be given to developing a sustainable technology implementation plan, one that anticipates rapid technological obsolescence and ensures long-term adaptability, fiscal responsibility, and curricular resilience. The decisions made in the coming years will shape the profession's curricula and hopefully bring about equity, sustainability, and continued societal relevance. A recent study underscored changes in educational practice to better align with the evolving expectations and learning habits of contemporary students. 2 The study found that all cohorts preferred virtual, interactive, case-based tutorials, perceiving them as somewhat more effective for learning than traditional in-person sessions, challenging the long-held assumption that learning must take place face-to-face. While preference does not equate to objective outcomes, it can indirectly boost effectiveness by enhancing motivation and self-directed learning. 6 Meta-analyses also show that blended approaches often outperform purely in-person or asynchronous formats, 7 8 suggesting that balancing structure with flexibility optimizes both engagement and performance. Fundamentally, cognitive psychology and active learning research indicate that effectiveness depends less on the medium and more on alignment with core learning principles—such as active engagement, timely feedback, and cognitive load management. 9 10 11 This invites further research into developing optimized, blended, and digitally enhanced educational models that preserve human connection while advancing accessibility and innovation, grounded firmly in established pedagogical theory to increase effectiveness without diminishing relational depth.

The integration of mixed-reality (MR) haptic-based dental simulators into preclinical education is a pedagogical necessity rather than just a technological enhancement. 12 13 Contemporary dentistry requires high levels of psychomotor precision, spatial awareness, and tactile discrimination—competencies that cannot be sufficiently developed through theoretical instruction alone. 14 15 MR haptic-based dental simulators provide calibrated force feedback, real-time performance metrics, and repeatable scenarios. 16 17 They establish a structured bridge between cognitive understanding and clinical execution, reducing variability and risk during early clinical training. 12 13 18 Furthermore, AI-driven tools, such as automated image analysis in orthopantomography, are emerging to advance diagnostic training, enabling students to interpret complex radiographs with greater accuracy and efficiency. 19 20 The integration of AI into electronic health record progress notes suggests that, with continued use, AI-assisted documentation may enhance perceived workflow efficiency and enable students and faculty to devote more focused, meaningful time to patient engagement. 21

## From Traditional Training to Immersive Ecosystems

For decades, phantom heads, benchtop exercises, and mentor-led clinical teaching have reliably nurtured dental students' psychomotor skills and professional identity. 21 22 23 According to contemporary pedagogical theories, learning materials should be accessible and adaptable to meet the needs of diverse students. 24 Learner-centered methods are also often required. Immersive technologies, such as haptic VR (HVR), augmented reality (AR), MR, AI-enhanced simulation, and virtual standardized patients (VSPs), are particularly suitable for this approach. 25 Integrating AI into these ecosystems, for instance, through generative models that evaluate student responses in licensing examinations, can further personalize learning and assessment, ensuring that educational tools evolve with technological advancements. 26 27 These technologies are already steadily redefining what preclinical and clinical training can achieve. A recent international survey confirms that classical methods still dominate—accounting for roughly four-fifths of clinical training time—while immersive modalities represent only a small fraction of activity. 28

Crucially, the dental profession is no longer debating whether immersive simulation belongs in curricula. The discussion has moved on to how to implement it sustainably, fairly, and at scale across vastly different economic and cultural contexts. 29 30 External constraints—such as funding, space, time, and structured faculty training—now overshadow internal resistance, signaling that the biggest obstacles are structural and political, not conceptual.

## Hybrid Simulation as the Emerging Gold Standard

While digital technologies may eventually support a large portion of dental students' hand-skill training, the hybrid educational model currently seems to be the most promising path. 31 32 Qualitative findings indicate that combining phantom heads, benchtop exercises, real-life materials, role play, HVR environments, and VSPs into deliberately designed hybrid models can preserve the strengths of traditional training while unlocking the full potential of immersive tools ( Fig. 1 ). 33 34 Hybrid simulation should be understood not as a temporary compromise, but as a framework for competency-based progression. 28 It enhances not only psychomotor skills but also spatial anatomical understanding and virtual interprofessional collaboration.

The digital transformation of dental education must become more than a technological upgrade; it should catalyze reimagining the very foundations of learning. 35 Everything that underpins dental learners' professional identity should be covered, including histology and anatomy, as well as biomedical and behavioral sciences. 36 Pre- and post-COVID studies on gamification in clinical reasoning and gamified virtual microscopy have shown that well-designed digital methodologies can do more than transfer lectures to a screen. 37 They can ignite curiosity, personalize learning, and cultivate deeper cognitive engagement. When technology is purposefully integrated, it can transform passive absorption into active exploration. 38 This helps students not just to memorize information but also discover understanding—the essence of education itself. Moreover, the increasing use of AI applications in dentistry, such as chatbots for question evaluation, can supplement hybrid models by providing instant feedback and reinforcing knowledge retention. 26 27 Importantly, digital transformation should also embed adaptive learning analytics that monitor student performance in real-time, enabling data-informed curricular refinement and early academic support to strengthen competency-based progression.

Furthermore, the cognitive and behavioral profiles exhibited by students during simulation-based training underscore its intrinsic pedagogical value. 39 40 Students working with haptic simulators exhibit heightened concentration, sustained attention, and deliberate practice behaviors that are often more constrained in live clinical settings. Immediate multimodal feedback and the absence of patient-related time pressures promote deep focus, procedural memory formation, and error recognition. 41 42 These are all foundational elements of safe and efficient clinical performance. Importantly, such focused practice cultivates professional confidence grounded in competence rather than trial and error. Ethical considerations in deploying AI for remote oral health monitoring further emphasize the need for student training on equitable integration, particularly among vulnerable populations, to avoid exacerbating disparities in access to education and care. 43 44

## Mesoscopic Anatomy and Multimodal Innovation

Traditionally, microanatomy (histology) and macroanatomy (gross structures) are addressed separately. Mesoscopic anatomy lies between these arenas, providing an intermediate level that can enhance understanding of health and disease. 45 46 Emerging simulator technologies—AR/VR/MR platforms, Sectra, VHDissector, and handheld wireless ultrasound probes, such as Vscan Air—offer detailed, accurate anatomy for improved learning that can better translate into safer clinical skills. 47 48

When simulations are embedded in problem-based and case-based learning, they evolve from peripheral add-ons into the backbone of longitudinal competence development. 49 50 However, any incorporation of new modalities should align with competency frameworks and be integrated with robust assessment systems. In such properly constructed ecosystems, AI-driven feedback may offer each learner a highly skilled “digital mentor,” supporting repetition, reflection, and individualized progression while upholding academic integrity. 51 52 For example, combining simulated underserved patients within communities with AI-powered mobile health (mHealth) tools for gingivitis detection and self-monitoring can extend this mentorship beyond the classroom, fostering preventive dentistry skills in real-world settings. 53 54 55 56

## Equity, Sustainability, and Global Networks

To address global challenges also means confronting inequity. Wealthier countries and higher-ranked institutions can integrate immersive tools earlier and more extensively. 57 58 This has raised concerns that digital transformation could widen gaps in graduate readiness and access to oral health care within and between regions. 59 60 61 Cross-institutional collaboration, shared infrastructure, pooled procurement, and global benchmarking can help democratize access to advanced simulation and align standards. 62 63 64 65 The role of non-governmental organizations (NGOs) in oral health promotion highlights how collaborative networks can leverage digital tools to address these inequities, particularly in underserved areas. 66 67 For example, systematic reviews of mHealth interventions to improve patients' oral health knowledge and behaviors demonstrate that these technologies, when properly applied, can enhance lifelong learning and preventive care. 68 69 Establishing open-access digital repositories, shared virtual simulation hubs, and transnational faculty development initiatives can further mitigate disparities by reducing duplication of costs and fostering collective capacity-building across institutions.

Networks such as the American Dental Education Association (ADEA), 70 the Association for Dental Education in Europe (ADEE), 71 and the Haptic and AI in Dental Education Network (Haptic and AI Digital Education Network for Excellence and ReSearch [HAIDENERS]) 72 are examples of what this type of global mindset can achieve. Efforts to foster distributed “niches” of innovation are collectively reshaping dental education culture while promoting equitable outcomes despite unequal resources. Active, multilevel communication with global associations such as the International Association for Dental, Oral, and Craniofacial Research (IADR) and dental equipment and tool manufacturers is essential. 73 74

Sustainability adds another compelling dimension ( Fig. 2 ). Traditional preclinical training often generates substantial material waste. 75 76 Conversely, VR-haptic simulators enable repeated practice without depleting physical resources. This shift could reduce both costs and environmental footprint while expanding access. 29 31 77 This aligns with the UN 2030 Agenda Sustainable Development Goals (SDGs) on responsible consumption and production, which focus on reducing waste generation through prevention, reduction, recycling, and reuse. 78 Additionally, AI in tooth reconstruction and edentulism rehabilitation can optimize resource use by enabling precise, customized educational simulations that minimize trial and error in training and support efficient rehabilitation treatment within communities. 79 80 81 82

## Regulation, Validation, and the Next Generation of Educators

Regulatory frameworks are beginning to acknowledge this shift. In several regions, knowledge of digital dentistry and its safe application is now an explicitly required element of basic dental training. 83 84 At the same time, rapid technological change and the risk of data drift—namely, changes in data inputs, meaning, or outcomes over time—make ongoing validation essential. 85 86 This underscores the need for a neutral global body to mediate between schools and industry and credential emerging technologies.

Perhaps most inspiring is the emergence of digitally native educators who are fluent in interactive platforms, immersive environments, and data-driven feedback. By mentoring colleagues, leading pilot projects, and contributing to multicenter research, they can transform local enthusiasm into collective evidence and policy change, ensuring innovation remains inclusive and educationally sound. Meta-research on instruments assessing oral health-related quality of life in older adults can guide these educators in incorporating AI to evaluate holistic outcomes in curricula. 87

## Ethical Issues in Preclinical Dental Simulation

From an ethical and deontological standpoint, it is increasingly difficult to justify exposing patients to procedures performed by novices who have not first demonstrated competence in simulated environments. 12 88 89 90 The principle of non-maleficence—the obligation to avoid causing harm—demands that dental education prioritize patient safety alongside student learning. Allowing students to initiate operative procedures directly on patients without extensive preclinical validation conflicts with this obligation, as it knowingly introduces preventable risk. 91 92 Simulation-based mastery learning reframes competence as a prerequisite rather than a byproduct of clinical exposure. 93 94 This ensures that first encounters with patients occur only after students have achieved objectively measured proficiency. In this sense, preclinical dental simulation becomes a moral imperative.

## Barriers and Challenges in Digital Transformation of Dental Education

While the integration of digital technologies such as AI, VR, and haptic simulation offers transformative potential for dental education, several limitations and challenges must be acknowledged to ensure balanced implementation. First, the high initial costs associated with acquiring and maintaining advanced simulators, including hardware, software licenses, and faculty training, pose significant barriers, particularly for institutions in low- and middle-income countries (LMICs). 28 95 96 These financial constraints can exacerbate existing inequities, creating a digital divide in which only well-resourced programs can fully adopt hybrid models, potentially widening gaps in graduate competencies and global oral health outcomes. 97 98 The implementation of technology in dental schools requires not only substantial capital investment but also structured faculty calibration, technical support, and phased curricular integration to ensure meaningful educational return on investment rather than superficial technological adoption. 99

Second, evidence on the long-term efficacy of these technologies remains limited. 39 Although short-term studies demonstrate improvements in psychomotor skills and student motivation, there is a paucity of longitudinal research evaluating how simulation-based training translates to sustained clinical performance, reduced error rates in practice, or improved patient outcomes over time. For instance, while HVR shows promise in preclinical settings, randomized controlled trials comparing hybrid approaches to traditional methods may not report equal results. Additionally, AI-driven tools, such as automated diagnostic aids, are susceptible to data drift—where model accuracy degrades due to evolving data inputs or demographic shifts—necessitating continuous validation that many educational institutions lack the expertise or resources to perform. 85

Third, faculty resistance and the need for upskilling represent human-centered challenges. 28 Many educators, trained in traditional paradigms, may lack familiarity with digital tools, leading to inconsistent integration and suboptimal use in curricula. This is compounded by ethical concerns, including data privacy in AI applications and the risk of overreliance on simulations, which could diminish real-world tactile sensitivity or interpersonal skills if not balanced with clinical exposure. 43 99 Cultural and contextual factors also play a role: For example, in diverse global settings, technologies may not account for variations in oral health needs or learning styles, potentially alienating non-digital-native learners. 84 100

Finally, sustainability extends beyond material waste reduction; the environmental impact of digital infrastructure, such as energy consumption from data centers supporting AI and VR, must be considered alongside benefits like reduced plastic use in phantom heads. Addressing these challenges requires rigorous, multicenter research to build a stronger evidence base, targeted funding for equitable access, and collaborative frameworks to mitigate biases and ensure inclusive adoption.

## Reimagining Dental Education for Better Patient Care

Over the past decade, generational shifts in digital fluency have increased student enthusiasm for VR and simulation, even as both older and newer cohorts agree that virtual tools should supplement rather than replace traditional methods. 33 Global dentistry now has an opportunity to lead not only in clinical science but in educational vision ( Fig. 1 ).

**Fig. 1:**
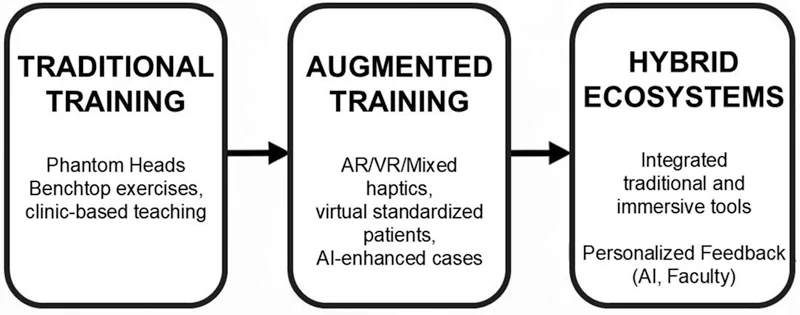
Evolution from traditional training to augmented and hybrid, digitally enhanced educational ecosystems in dental education. AI, artificial intelligence; AR, augmented reality; VR, virtual reality.

The combined adoption of MR haptic-based simulators is driven by the overarching goal of measurably improving clinical practice. 101 102 103 104 These educational modalities are primarily means through which higher standards of care can be systematically achieved. By enabling students to internalize precise motor skills, refine clinical judgment, and develop error awareness before treating patients, simulation-based training contributes to more predictable, efficient, and biologically respectful procedures. As graduates enter clinical practice with a deeper reservoir of validated competencies, the cumulative effect is reflected in the quality of their work: Fewer iatrogenic complications, more durable restorations, and more consistent treatment outcomes. In this context, the ultimate measure of success in dental education—and in dentistry as a profession—lies in its capacity to enhance patients' quality of life ( Fig. 2 ). 89

**Fig. 2:**
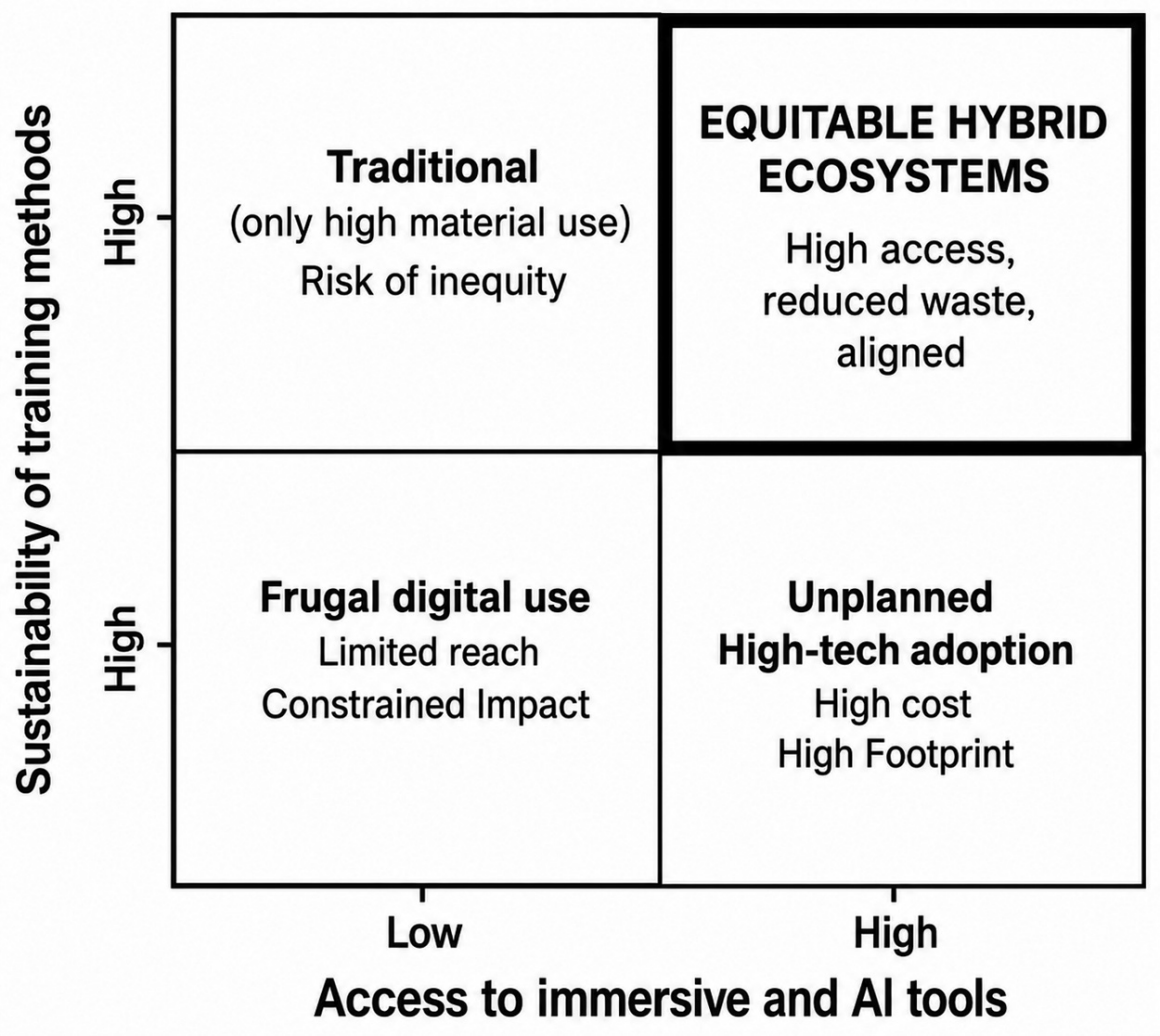
Conceptual matrix illustrating the relationship between access to immersive technologies, sustainability, and the risk of a digital divide in dental education. AI, artificial intelligence.

## Call to Action: Building a Collaborative Future for Dental Education

The time for incremental change has passed; dental education must embrace a bold, collective vision to harness digital technologies for equitable, sustainable, and patient-centered outcomes. Educators, regulators, professional associations (e.g., ADEA, ADEE, IADR, HAiDENERS), and industry partners are called upon to prioritize the following actions ( Fig. 3 ):

**Fig. 3:**
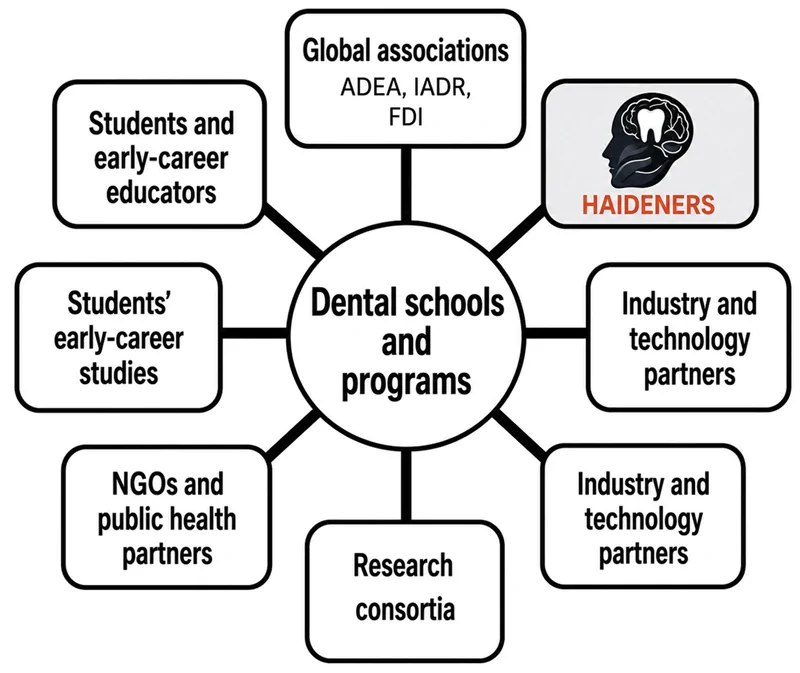
Multilevel collaboration among dental schools, associations, NGOs, regulators, and industry to support equitable, validated digital transformation. ADEA, American Dental Education Association; IADR, International Association for Dental, Oral, and Craniofacial Research; NGO, non-governmental organization.

1. **Foster global collaborations** : Establish international consortia for shared resource procurement, benchmarking standards, and knowledge exchange to democratize access to HVR, AI, and hybrid simulation tools, particularly in underserved regions. This includes funding open-source platforms and pilot programs in LMICs to bridge the digital divide.
2. **Invest in evidence-informed research** : Launch multicenter, longitudinal studies to evaluate the real-world impact of digital integrations on clinical competence, patient safety, and educational equity. Regulators should mandate ongoing validation of AI tools to address data drift and biases, ensuring ethical deployment.
3. **Enhance faculty development** : Develop comprehensive training programs for digitally native educators to mentor peers, integrating competencies in VR/AR and AI into accreditation standards. This will cultivate a cadre of innovators capable of reimagining curricula while preserving core humanistic elements.
4. **Align with sustainability goals** : Commit to eco-friendly practices by prioritizing low-waste digital simulations and advocating for policies that support UN SDGs, including lifecycle assessments of educational technologies.

By acting decisively, dental education can be transformed into a resilient, inclusive ecosystem that prepares graduates for a digital future and advances global oral health equity. Let us seize this pivotal moment to lead, innovate, and collaborate—for the benefit of learners, professionals, and patients worldwide.

This communiqué was co-created by the undersigned authors, who contributed as speakers and participants during the HAiDENers Hybrid Summit 2026, held in February 2026 at Planmeca Headquarters in Helsinki, either on-site or online. The meeting showcased the evolution of digital dental education driven by academic collaboration and technological innovation ( Fig. 3 ), building on more than five decades of Planmeca's partnership with universities and clinicians in developing and validating advanced simulation and imaging solutions. Scientific sessions led by Professors Sompop Bencharit (UNC, NC, United States), ADEA President and CEO Dr. Karen P. West, Hiroe Ohyama (Harvard), Andreea Didilescu (Bucharest, Romania), Damiano Pasqualini (Turin, Italy), and other international experts highlighted the importance of interdisciplinary cooperation in preparing future-ready oral health professionals.
